# Neurotrophin-3 (NT-3) as a Potential Biomarker of the Peripheral Nervous System Damage Following Breast Cancer Treatment

**DOI:** 10.3390/pathophysiology30020010

**Published:** 2023-04-03

**Authors:** Samvel Tonyan, Maria Pospelova, Varvara Krasnikova, Olga Fionik, Tatyana Alekseeva, Konstantin Samochernykh, Nataliya Ivanova, Tatyana Vavilova, Elena Vasilieva, Albina Makhanova, Aleksandra Nikolaeva, Tatyana Bukkieva, Stephanie Combs, Maxim Shevtsov

**Affiliations:** 1 Personalized Medicine Centre, Almazov National Medical Research Centre, 2 Akkuratova Str., 197341 Saint Petersburg, Russia; samvelium@gmail.com (S.T.); pospelovaml@mail.ru (M.P.); varya.krasnikova.93@mail.ru (V.K.); fvolga@mail.ru (O.F.); atmspb@mail.ru (T.A.); neurobaby12@gmail.com (K.S.); ivamel@yandex.ru (N.I.); vtv.lab.spb@gmail.com (T.V.); elena-almazlab@yandex.ru (E.V.); a.mahanova.a@mail.ru (A.M.); shura.nicolaeva@yandex.ru (A.N.); tanya-book25@mail.ru (T.B.); 2 Department of Radiation Oncology, Technishe Universität München (TUM), Klinikum Rechts der Isar, Ismaninger Str. 22, 81675 Munich, Germany; stephanie.combs@tum.de

**Keywords:** peripheral nervous system, neuropathy, postmastectomy pain syndrome, chemotherapy-induced peripheral neuropathy, radiation-induced peripheral neuropathy, brain-derived neurotrophic factor, BDNF, neurotrophin-3, galectin-3, breast cancer, mastectomy

## Abstract

Damage to the peripheral nervous system (PNS) is a common complication of breast cancer (BC) treatment, with 60 to 80% of breast cancer survivors experiencing symptoms of PNS damage. In the current study, the levels of brain-derived neurotrophic factor (BDNF), galectin-3 (Gal-3), and neurotrophin-3 (NT-3) were measured in the blood serum of BC patients by ELISA as potential biomarkers that might indicate the PNS damage. Sixty-seven patients were enrolled in this multi-center trial and compared to the aged-matched healthy female volunteers (control group) (*n* = 25). Intergroup comparison of biomarker levels (i.e., Gal-3 and BDNF) did not show significant differences in any of the studied subgroups. However, intriguingly, NT-3 levels were significantly higher in BC patients as compared to healthy volunteers, constituting 14.85 [10.3; 18.0] and 5.74 [4.56; 13.7] pg/mL, respectively (*p* < 0.001). In conclusion, NT-3 might be employed as a potential biomarker in BC patients with clinical manifestations of PNS damage. However, further studies to validate its correlation to the degree of peripheral nervous system lesions are of high value.

## 1. Introduction

Breast cancer (BC) represents the most common type of cancer. Indeed, in 2020 alone, more than 2.3 million breast cancer cases were detected [1]. Furthermore, population growth and aging are projected to increase the incidence and consequences of breast cancer treatment, especially in low- and middle-income countries [2].

Early detection of breast cancer and comprehensive treatment can lead to a 90% chance of survival. Currently, BC treatment protocol includes surgery [3], radiation therapy [4], and chemotherapy [5]. Depending on tumor characteristics and its spread, the appropriate method or combinations thereof are determined. Cancer therapy also includes hormone therapy and, in some cases, targeted biological therapy [6]. Unfortunately, the radical nature of most of the described methods of treatment leads to a decrease in the quality of life of breast cancer survivors [7,8].

Radical components of breast cancer treatment damage the peripheral nervous system (PNS). Several studies demonstrated that 60% of patients following breast cancer surgery manifested persistent pain associated with the younger age, complexity of therapy, axillary lymph node dissection, and high preoperative pain [9].

Radiation-induced peripheral neuropathy (RIPN), in most cases, is clinically asymptomatic. Usually, the symptoms manifest with hypoaesthesia or dysaesthesia and can further progress to anaesthesia. However, in rare cases, neuropathic pain was observed [10].

Chemotherapy-induced peripheral neuropathy (CIPN) is a common consequence of chemotherapy, especially when a taxane group of agents is employed, reaching the frequency of occurrence in breast cancer survivors of up to 63%. This complication is dose-dependent, affects the entire nervous system, and is manifested by symptoms of polyneuropathy, including symmetrical disturbance of sensitivity, tingling, a violation of fine motor skills, and sometimes pain in the extremities [11,12,13].

Generally, patients experience chronic pain and impaired sensitivity in the armpit, chest, and upper limbs. This symptomatology is usually termed postmastectomy pain syndrome (PMPS) [14,15].

Currently, rare studies are devoted to identifying biomarkers of PNS damage in women who survived breast cancer. In addition, few works analyze the polymorphism of genes that may be associated with chronic pain syndrome after breast cancer [16,17,18].

Specific biomarkers of PNS lesions in patients following BC treatment have not yet been identified. However, according to the available literature data, among many potential biomarkers, neurotrophin-3 (NT-3) [19], brain-derived neurotrophic factor (BDNF) [20] and galectin-3(Gal-3) [21] could be used as possible markers of the PNS damage.

Neutrophin-3 (NT-3) is expressed in the brain, PNS and other tissues, including the heart, liver, pancreas, and kidneys. NT-3, like BDNF, Nerve Growth Factor (NGF), and Neutrophines-4-6, belongs to the neurotrophin family [22]. In a developing organism, NT-3 establishes synaptic contact through stimulation of axon growth [23,24]. Furthermore, NT-3 promotes the survival and differentiation of existing neurons and the growth and differentiation of new neurons [25]. Several preclinical studies showed the therapeutic potency of NT-3 in peripheral nerve repair [26,27,28]. Thus, Gao et al. showed that fibronectin mats impregnated with NT-3 and implanted in rats into a 10 mm gap of an injured sciatic nerve significantly increased the number of myelinated axons that was comparable to the NT-3 levels of the control group with intact sciatic nerve [29]. In another study, modulation of muscle pain by NT-3 was demonstrated in a model of mechanical hyperalgesia caused by acid injection in both paws of wild-type mice. Indeed, exogenous, and overexpressed endogenous neurotrophin-3 significantly reduced the duration of secondary hyperalgesia and the likelihood of the process becoming chronic. Of note, the neuroprotective effect of NT-3 was detected only when the protein was applied in the acute phase of damage [30]. Another study by Wilson-Gerwing et al. showed that intrathecal administration of NT-3 significantly attenuates the expression of nociceptive sodium channels involved in the formation of neuropathic pain syndrome in chronic compression injury, reinforcing the role of the analgesic effect of neurotrophin-3 [31]. NT-3 also mediates neuroprotection by increasing the survival rate of Schwann cells and regulating the oligodendrocytes level [32,33].

Brain-derived neurotrophic factor (BDNF) is among the most abundant and widely studied neurotrophins [34]. BDNF supports the survival of emerging neurons and increases the number and differentiation of new neurons and synapses [35]. BDNF is involved in central sensitization and synaptic plasticity in the brain and spinal cord [36]. This biomarker has been shown to promote the development and maintenance of neuropathic pain by activating NR2B-containing NMDA receptors (NMDA-2B) in the spinal cord’s dorsal horns [37,38]. Huang et al. showed that conditional knockout of BDNF from microglia of mice with peripheral nerve injury prevented pain hypersensitivity [39]. The occurrence of neuropathic manifestations in PMPS may result from this marker’s maladjustment effect [40]. One of the proposed mechanisms of action of BDNF in neuropathic pain may be the increased sensitivity of neurons to pain stimuli and increased co-expression of thermo-TRP channels [41]. The study of Marcol et al. demonstrated that local inactivation of BDNF in sciatic nerves of adult male rats with anti-BDNF antibodies decreases the severity of nerve degeneration. On the contrary, in studies where BDNF was used as a therapeutic agent, a significant neuroprotective effect was observed [42,43,44].

Galectin-3 (Gal-3) plays a significant role in cell adhesion, cell activation and chemoattraction, cell growth and differentiation, cell cycle, and apoptosis. Gal-3 has been identified in macrophages and tissues of the heart, liver [45], and kidneys [46], correlating with various fibrosis types [47,48]. Up-to-date, the potential role of Gal-3 as a biomarker for PNS damage was described in several studies [49,50]. Thus, Ma et al. noted that dedifferentiated Schwann cells show high expression of galectin-3 in anterograde degeneration following peripheral nerve injury, which plays an important role in the lectin-mediated phagocytosis of degraded material at the injury site [21]. It has also been demonstrated that inhibition of Gal-3 can suppress neuroinflammation, alleviate neuropathic pain caused by damage to peripheral nerves, and accelerate the recovery of nervous tissue [51]. Koyanagi et al. hypothesized that Schwann cell galectin-3, released into the extracellular compartment, could be involved in the pathogenesis of CIPN by dedifferentiation and mitochondrial dysfunction, particularly following the chemotherapy with the taxane group agents [52].

Most of the aforementioned scientific papers reported the results of preclinical studies that involved various animal models. Currently, few studies attempted to identify the plausible biomarkers of PNS damage in women who survived breast cancer, thus indicating the necessity of such trials.

In the current study, we explored the possibility of employing BDNF, NT-3 and galectin-3 as biomarkers to identify lesions of the peripheral nervous system in women following breast cancer treatment.

## 2. Materials and Methods

### 2.1. Experimental Design

The study was conducted per the principles of the Helsinki Declaration of the World Medical Association with the consent of the Ethics Committee of the Federal State Budgetary Institution “Almazov National Medical Research Center” of the Ministry of Health of the Russian Federation (conclusion of 24 January 2022).

#### 2.1.1. Inclusion Criteria

The recruitment is accomplished from the database of patients who received breast cancer treatment in oncology centers in St. Petersburg (Russia) from 2012 to 2023. The main selection criteria included the age from 25 to 50 years, the period after surgery of more than six months, and the absence of concomitant diseases that prevent examination. The control group of women was collected from healthy volunteers in the same age range with no symptoms of PNS damage or severe somatic diseases. All women signed written informed consent to participate in the study.

#### 2.1.2. Exclusion Criteria

Exclusion criteria: signs of breast cancer recurrence; identified distant metastases of breast cancer; the pregnancy period; the final score of more than 45 on the Spielberg-Khanin scale of reactive and personal anxiety; acute musculoskeletal injuries [53].

### 2.2. Neurological Examination

Patients were examined in the morning on an outpatient basis. A detailed anamnesis of breast cancer was collected, including data on the date of the disease, the TNM stage [54], data on the courses of chemotherapy and radiation therapy, and the subsequent intake of anticancer drugs (Tamoxifen^®^ (Sandoz, Basel, Switzerland), Herceptin^®^ (Hoffmann-La Roche Ltd., Basel, Switzerland)).

All participants in the study underwent a neurological examination and collection of complaints. Muscle strength was assessed using the Medical Research Council (MRC) muscle scale. Gait, posture, and range of motion in the limbs were also assessed. Pain sensitivity was evaluated in patients with complaints of numbness and reduced sensitivity in the arm compared to the contralateral limb or in a more proximally/distally located arm area. Pain sensitivity was assessed with the help of pricks with the sharp end of a toothpick on symmetrical areas of the face, limbs, and torso, followed by disposal of the toothpick. An objective examination assessed the type of sensitivity disorders: neural, polyneuritic, and radicular. Before testing, patients were introduced to the examination methodology. We asked patients to close their eyes and describe their sensations during testing: decrease or increase in the injection, a complete absence of sensations, whether it feels sharp or dull, single or multiple, does it cause pain. All patients’ responses were fixed on a schematic image of the body. The level and nature of the lesion were determined topically. Hypesthesia was identified when patients felt a less intense or dull prick compared to the healthy side.

Cold sensitivity was determined in a calm environment, at a comfortable temperature (22 °C), in a quiet room. Then, a test tube with cold water was applied to the symmetrical points. At the same time, the patient’s subjective sensation of cold and the ability to differentiate temperatures were evaluated.

Assessment of deep sensitivity was carried out by determining the joint-muscular feeling. The study was carried out with the eyes of the patients closed. Previously, we agreed with patients which direction of movement would mean “up” and which “down”. Next, we asked them to determine the direction of passive movements in the joints of the arms and legs. First, we performed passive movements in small and larger joints. Violation of the articular-muscular feeling was fixed if the patients incorrectly named the finger or the direction of its passive movement.

Vibration sensitivity was tested using a tuning fork vibrating at 128 Hz on the back of the distal interphalangeal joint. The examiner’s finger was on the inside of the joint. The difference with which the doctor and the subject cease to feel the vibration is determined. A sensitivity assessment was carried out symmetrically in patients with their eyes closed. Sensitivity in the axillary zone, the area of the shoulder blades, and the chest were examined. The intensity of the pain syndrome was assessed using a visual analog scale (VAS) for pain.

### 2.3. Enzyme-Linked Immunosorbent Assays

The 7 mL venous blood serum was taken from all participants by venipuncture with a butterfly needle and collected in Becton Dickinson serum separator tubes (SSTs) after centrifugation was aliquoted and stored at −80 °C in biobank conditions.

The levels of neurotrophin-3, galectin-3 (ELISA-Kit, Elabscience Biotechnology Inc., Houston, TX, USA), and brain-derived neurotrophic factor (ELISA-Kit, R&D Systems, Minneapolis, MN, USA) were determined using enzyme-linked immunosorbent assay. The analysis was carried out according to the manufacturer’s protocol.

### 2.4. Statistical Analysis

Statistica 12.5 software (TIBCO Software Inc., Palo Alto, CA, USA) was used for statistical analysis. We used absolute and relative indicators of the number of observations to assess qualitative variables. *p*-values less than 0.05 are considered statistically significant.

The normality of the distribution was assessed using the Shapiro-Wilk test. Statistical hypotheses were tested using the Kruskell-Wallis test. Finally, post hoc analysis was performed in pairs using the Mann-Whitney U-test for groups demonstrating a statistically significant result.

## 3. Results

### 3.1. Patients

The study group included 67 Caucasian women aged 30 to 50, with an average age of 47 (44, 49) years, with complications from radical breast cancer treatment. The group of healthy volunteers included 25 Caucasian women with an average age of 42 (38, 47) years. The average time elapsed after surgery constituted 3 (2, 5) years. In 68% (*n* = 46) of patients, the disease was detected at the T2 stage with no distant metastasis. The most common form of BC was invasive ductal carcinoma (73%, *n* = 49). All patients underwent surgical treatment, and 55% (*n* = 37) of women underwent complex therapy. In 79% (*n* = 53) of cases, the operation of choice was Madden-modified radical mastectomy. Only 7% (*n* = 5) took, and 15% (*n* = 12) of patients continue to take hormone therapy (Table 1).

All patients were divided into groups depending on PNS lesion symptoms. The largest number (*n* = 46, 69%) of women was included in the group with PMPS (Table 2).

According to the VAS, the average pain level constituted 4 (2.5, 5.25) points. Checking the level of temperature sensitivity and joint-muscular feeling demonstrated the absence of defects in these types of sensitivity in patients. Vibration sensitivity in all cases was symmetrically impaired in the distal extremities. In 76% of patients with a violation of the vibration sense, there was a history of chemotherapy for breast cancer. At this rare impairment, vibration sensitivity was used to identify patients with polyneuropathy hypesthesia.

### 3.2. Evaluation of BDNF, NT-3, and Gal-3

The serum level of biomarkers in breast cancer survivors and the control group was compared. The level of NT-3 (16.62 [11.18; 20.0] pg/mL) was 3-fold higher than in the control group (5.74 [4.56; 13.7] pg/mL) (*p* < 0.001). On the other hand, the levels of BDNF (31747.4 [23,068.0; 37,903.0] pg/mL) and Gal-3 (29,281.6 [21,786.4; 35,728.2] ng/mL) did not differ significantly in breast cancer survivors and healthy volunteers, constituting 29,281.6 [21,786.4; 35,728.2] and 4660.0 [3240.0; 6380.0] ng/mL, accordingly (Table 3).

In the intergroup comparison of biomarker levels, galectin-3 and BDNF did not show significant differences in any of the studied subgroups. However, the NT-3 significantly varied within each of the subgroups. In almost every group, the level of neurotrophin-3 was significantly lower than in the group of healthy volunteers. The serum levels of detected biomarkers and statistical results of the analysis are presented in Table 4.

**Figure 1:**
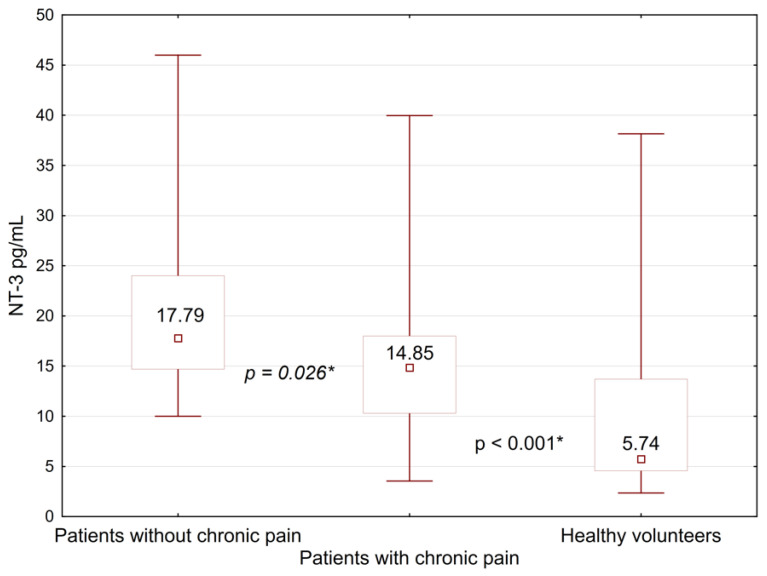
Comparison of neurotrophin-3 levels between women with PMPS, its absence, and a group of healthy volunteers. *—differences between the groups were significant at *p* < 0.05.

A comparison of the NT-3 was carried out within each of the subgroups. Serum level of NT-3 in patients with PMPS (14.85 [10.3; 18.0] pg/mL) was significantly lower (*p* = 0.026) than in patients with no PMPS symptoms (17.79 [14.7; 24.0] pg/mL) (Figure 1).

NT-3 levels in both subgroups were significantly higher than in healthy volunteers (*p* < 0.001). In all other cases, no statistically significant differences were detected between the subgroups. The level of neurotrophin-3 in the healthy group was significantly reduced compared to all subgroups. Detailed information on the level of neurotrophin-3 in subgroups is presented in Table 5.

## 4. Discussion

The quality of care for women with breast cancer is steadily increasing [55,56]. However, complications of breast cancer treatment occur in 80% of cases [57]. One of the clinical variants is neuropathic manifestations, in which damage to the peripheral nervous system occurs due to compression (lymphedema), radiation, or chemotherapy [58].

The study of biomarkers of PNS lesions has been ongoing for a long time. There is enough evidence in the scientific literature to conclude that studying specific biomarkers could help diagnose PNS lesions. However, only a few works are devoted to studying these markers in patients with neuropathy symptoms following breast cancer treatment [59,60,61,62,63,64,65].

In the current study, we analyzed biomarkers with a supposed role in reflecting the state of the affected PNS in BC survivors. Gal-3 was chosen for the study due to data on its role in lectin-mediated phagocytosis in peripheral nerve fibers damage and activation of inflammation during neurodegeneration [66]. However, the level of Gal-3 in studied patients constituted 5450.0 [4080.0; 9900.0] ng/mL and did not differ significantly from the group of healthy volunteers (4660.0 [3240.0; 6380.0] ng/mL) with *p* = 0.26. Based on the presented data from Koyanagi et al. (2021), we expected to detect changes in galectin-3 in patients in the group with CPIN symptoms [52]. However, the level of gal-3 in this group was 5090.0 [4070.0; 8760.0] ng/mL and did not differ significantly from the level of this marker in patients without spinal symptoms (6340.0 [4620.0; 9900.0] ng/mL) and in healthy volunteers (4660.0 [3240.0; 6380.0] ng/mL).

Interestingly, Fionik et al. (2021) showed breast cancer survivors are more likely to develop soft tissue fibrosis over the years. Therefore, future measurement of galectin-3 in patients with presumed fibrosis and PNS damage could be an appropriate study goal [67].

Based on the literature data, we predicted a significant change in the level of neurotrophins in patients with clinical manifestations of PNS damage [68,69]. The BDNF marker was supposed to also be used as a marker of brain involvement in the presented symptomological picture. Therefore, it was assumed that BDNF could demonstrate the relationship between PNS damage and the presence of pain [37,38,39]. However, the level of BDNF did not change significantly in any of the selected groups of patients. Serum BDNF level (31,747.4 [23,068.0; 37,903.0] pg/mL) in breast cancer survivors did not differ significantly from the level of this biomarker in healthy volunteers (29,281.6 [21,786.4; 35,728.2] pg/mL) with *p* = 0.33.

In works studying the role of BDNF in the development of neuropathy, the idea of the involvement of the higher cortex as one of the main fields of influence of this protein was traced [42,43]. Therefore, it could be assumed that after breast cancer treatment, women do not have functional or structural changes in the brain. On the contrary, in the work of Bukkieva et al. (2021), using functional MRI, the presence of changes in the structural connectome of the brain in patients who survived breast cancer was demonstrated [70]. Also, the absence of significant changes in the level of BDNF between groups of patients may indicate a long period that has passed since the PNS lesion. However, most of the measurements were taken directly during nerve damage. Long-term measurements of BDNF in laboratory animals with damaged PNS could not be found. In addition, experimental models use a more severe peripheral nerve lesion than expected in women after breast cancer treatment.

The antinociceptive function of NT-3 has been actively studied in recent years [71,72,73,74]. In our study, NT-3 was the only one of the three biomarkers that showed a statistically significant result. Thus, NT-3 was significantly elevated in breast cancer survivors (16.62 [11.18; 20.0] pg/mL) relative to the volunteer group (5.74 [4.56; 13.7] pg/mL) with *p* < 0.001. To explain the differences in the levels of this biomarker, a comparison was made between groups of patients, divided depending on the prevailing symptoms or medical history. A statistical difference in the NT-3 between a group of volunteers and each of the selected groups of patients may be due to mutual overlap. Pain, as the main and most frequent symptom in this category of patients, was the only one that showed a significant result in its absence [14,15].

The level of the biomarker in women with manifestations of PMPS turned out to be higher than in the group of healthy women and amounted to 14.85 [10.3; 18.0] pg/mL with *p* = 0.001. This fact is consistent with the available literature data, in which a reduced level of NT-3 or its absence leads to reduced regeneration of peripheral nerve fibers. Or increased levels of NT-3 in laboratory models led to better recovery of the PNS. It can be assumed that the level of NT-3 is produced compensatory in response to the presence of damage to peripheral nerve fibers.

It appeared that the NT-3 was also statistically significantly increased in the group of women who had breast cancer but did not have pain due to PMPS (17.79 [14.7; 24.0] pg/mL). Interestingly, the biomarker level was significantly higher than in patients with pain with *p* = 0.026. Plausibly, the decrease in NT-3 levels in the pain group, relative to women without pain, resulted from depletion and/or underproduction of this biomarker. In addition, it should be noted that the level of NT-3, in this case, reflects not an acute but a chronic process.

Thus, it is impossible to assess the degree of PNS damage by the level of NT-3, but this marker presumably has the potential to indicate the lesion.

The limitations of this study include a small number of patients in separate subgroups and difficulties with the distribution of patients into different non-overlapping subgroups. Also, we cannot reliably compare pain intensity with the level of biomarkers due to the subjectivity of VAS.

Studied molecules cannot cover the entire spectrum of damage to the nervous tissue. However, each of the studied proteins can reflect a specific link in the chain of pathogenesis in PNS lesions. Therefore, it is necessary for future studies to assess the levels and degree of PNS damage in women after breast cancer treatment using instrumental diagnostic methods and correlate these findings to the candidate biomarkers of PNS lesions.

## 5. Conclusions

Among the studied biomarkers, the NT-3 was significantly increased (compared to healthy volunteers) in the blood serum of women who survived breast cancer and clinically manifested with chronic pain. Presumably, neutrophin-3 could be employed as a potential biomarker of PNS damage in BC patients following treatment. However, further studies to validate its correlation to the degree of peripheral nervous system lesions are highly important.

## Figures and Tables

**Table 1:** The number and percentage of study participants were divided into groups depending on the anamnesis.

Group Characteristic	Patients after BC Treatment (*n* = 67)
Age (years)	47 (44, 49)
Years after the end of therapy	3 (2, 5)
The structure by stages TNM, UICC	
I (T1N0M0)	8 (12%)
II A (T2N1M0)	46 (68%)
II B (T3N1M0)	3 (5%)
III A (T3N2M0)	2 (3%)
III B (T4N2M0)	8 (12%)
The histological type of breast cancer	
Ductal carcinoma in situ (DCIS)	7 (11%)
Invasive ductal carcinoma (IDC)	49 (73%)
Invasive lobular carcinoma (ILC)	11 (16%)
Type of breast cancer treatment	
Complex therapy of breast cancer (surgical treatment, radiation therapy, chemotherapy)	37 (55%)
Surgical treatment and chemotherapy	18 (27%)
Surgical treatment and radiation therapy	7 (10%)
Only surgical treatment	5 (7%)
Type of surgical treatment	
Madden-modified radical mastectomy	53 (79%)
Sectoral resection	14 (21%)
Hormone therapy (Tamoxifen ± GnRH analogue)	
Yes	12 (18%)
No	50 (75%)
Completed the course	5 (7%)

**Table 2:** Division into groups depending on the symptoms of PNS lesions.

Clinical Characteristics	Number of Patients (N, %)
Chronic pain syndrome	46 (69%)
Numbness in the armpit	45 (67%)
Polyneuropathy	34 (51%)

**Table 3:** Comparison of the level of biomarkers of breast cancer survivors and a group of healthy volunteers.

Biomarker	BC Survivors (*n* = 67)	Healthy Volunteers (*n* = 25)	Mann-Whitney U Test	Significance (*p*)
NT-3 pg/mL	16.62 [11.18; 20.0]	5.74 [4.56; 13.7]	254	<0.001 *
BDNF pg/mL	31,747.4 [23,068.0; 37,903.0]	29,281.6 [21,786.4; 35,728.2]	534.5	0.33
Gal-3 ng/mL	5450.0 [4080.0; 9900.0]	4660.0 [3240.0; 6380.0]	521.0	0.26

*—differences between the groups were significant at *p* < 0.05.

**Table 4:** Intergroup comparison of biomarker levels.

Sing of Separation	Characteristic of the Sing	Number of Patients (and Age)	NT-3 pg/mL	Kruskal–Wallis Test	*p*	BDNF pg/mL	Kruskal–Wallis Test	*p*	Gal-3 ng/mL	Kruskal–Wallis Test	*p*
Chronic pain syndrome	yes	46 (47 [43; 48])	14.85 [10.3; 18.0]	19.05	<0.001 *	32,291.2 [25,359.0; 39,417.4]	4.45	0.11	5600.0 [4160.0; 9140.0]	1.12	0.57
	no	21 (47 [40; 50])	17.79 [14.7; 24.0]			29,572.0 [20,621.0; 35,456.0]			6350.0 [3340.0;13,960.0]		
Healthy volunteers			5.74 [4.56; 13.7]			29,281.6 [21,786.4; 35,728.2]			4660.0 [3240.0; 6380.0]		
Hypoesthesia in the armpit	yes	45 (46 [40; 48])	16.0 [11.74; 20.0]	15.22	<0.001 *	31,378.0 [23,689.0; 37,903.0]	1.13	0.57	5200.0 [4060.0; 9980.0]	1.74	0.42
	no	22 (48 [44; 49])	17.03 [11.18; 20.0]			32,232.0 [22,757.2; 38,136.0]			6230.0 [4620.0; 9900.0]		
Healthy volunteers			5.74 [4.56; 13.7]			29,281.6 [21,786.4; 35,728.2]			4660.0 [3240.0; 6380.0]		
Polyneuropathy	yes	34 (46 [42; 48])	15.58 [11.75; 17.36]	16.4	<0.001 *	31,164.7 [22,601.5; 39,223.2]	1.11	0.57	5090.0 [4070.0; 8760.0]	1.53	0.47
	no	33 (48 [43; 49])	17.06 [11.18; 21.46]			32,077.0 [24,155.4; 36,815.0]			6340.0 [4620.0; 9900.0]		
Healthy volunteers			5.74 [4.56; 13.7]			29,281.6 [21,786.4; 35,728.2]			4660.0 [3240.0; 6380.0]		
Treatment history	Only surgical treatment	5 (44 [40; 48])	17.36 [14.18; 19.12]	17.03	0.0019 *	30,776.5 [18,225.0; 45,080.8]	1.87	0.75	4130.0 [3580.0; 6290.0]	2.99	0.55
	Surgical treatment and radiotherapy	7 (47 [46; 47])	16.0 [15.0; 18.82]			30,951.4 [28,038.0; 32,504.8]			6340.0 [5140.0; 7080.0]		
	Surgical treatment and Chemotherapy	18 (46 [42; 48])	17.35 [14.26; 22.35]			31,203.9 [21,320.1; 39,417.5]			6110.0 [4070.0; 8360.0]		
	Complex treatment	37 (46 [43; 49])	13.53 [10.0; 18.96]			32,386.8 [25,378.5; 37,320.0]			5350.0 [4220.0; 10,660.0]		
Healthy volunteers			5.74 [4.56; 13.7]			29,281.6 [21,786.4; 35,728.2]			4660.0 [3240.0; 6380.0]		
Type of surgery	Modified unilateral mastectomy Madden	53 (47 [43; 48])	16.88 [11.75; 20.0]	15.84	<0.001 *	32,174.7 [22,135.6; 38,407.5]	1.08	0.58	5420.0 [4120.0; 10,240.0]	1.30	0.52
	Sector mastectomy	14 (46 [41; 48])	15.0 [9.42; 19.4]			31,456.0 [25,359.0; 37,242.0]			6160.0 [4060.0; 8140.0]		
Healthy volunteers			5.74 [4.56; 13.7]			29,281.6 [21,786.4; 35,728.2]			4660.0 [3240.0; 6380.0]		

*—differences between the groups were significant at *p* < 0.05.

**Table 5:** Intragroup comparison of NT-3 levels.

Sing of Separation	Compared Groups	Mann-Whitney U Test	*p*
Chronic pain syndrome	Yes/No	264	0.026 *
	Healthy volunteers (Healthy)/No	44	<0.001 *
	(Healthy)/Yes	208	<0.001 *
Hypoesthesia in the armpit	Yes/No	463	0.90
	(Healthy)/No	79.5	<0.001 *
	(Healthy)/Yes	173.5	<0.001 *
Polyneuropathy	Yes/No	436	0.22
	(Healthy)/No	112.5	<0.001 *
	(Healthy)/Yes	140.5	0.001 *
Type of surgery	Modified unilateral mastectomy Madden (M)/Sector mastectomy (SM)	294	0.48
	(Healthy)/(M)	183	<0.001 *
	(Healthy)/(SM)	70	0.04 *
Breast cancer treatment	Only surgical treatment (OS)/Surgical treatment and radiotherapy (S + R)	9	0.90
	Surgical treatment and Chemotherapy (S + Ch)/Complex treatment (CT)	179	0.09
	(OS)/(S + Ch)	29	0.81
	(OS)/(CT)	50	0.50
	(S + R)/(S + Ch)	34	0.65
	(S + R)/(CT)	71	0.71
	(OS)/(Healthy)	11	0.03 *
	(S + R)/(Healthy)	26	0.014 *
	(S + Ch)/(Healthy)	46	<0.001 *
	(CT)/(Healthy)	139	0.001 *

*—differences between the groups were significant atp < 0.05. Healthy volunteers (Healthy), Only surgical treatment (OS), Surgical treatment and radiotherapy (S + R), Surgical treatment and Chemotherapy (S + Ch), Complex treatment (CT).

## Data Availability

The data presented in this study are available on request from the corresponding author. The data are not publicly available due to informed consent confidentiality paragraph.
